# Protocol for dynamic eye-brain imaging by integrating an eye-tracking system with magnetic resonance imaging

**DOI:** 10.1016/j.xpro.2026.104903

**Published:** 2026-10-27

**Authors:** Jaime Barranco, Yiwei Jia, Eleonora Fornari, Oscar Esteban, Jean-Baptiste Ledoux, Mauro Leidi, Helene Vitali, Cédric Campos Carvalho, Elena Najdenovska, Laura Elena Raileanu, Jessica Bastiaansen, Micah M. Murray, Benedetta Franceschiello

**Affiliations:** 1 HES-SO University of Applied Sciences and Arts Western Switzerland, Sion, Switzerland; 2 The Sense Innovation and Research Centre, Lausanne, Switzerland; 3 Department of Radiology, Lausanne University Hospital and University of Lausanne, Lausanne, Switzerland; 4 Graduate School for Cellular and Biomedical Sciences, University of Bern, Bern, Switzerland; 5 CIBM Center for Biomedical Imaging, Lausanne, Switzerland; 6 Unit for Visually Impaired People, Italian Institute of Technology, Genova, Italy; 7 Department of Computer Science, Bioengineering, Robotics and Systems, University of Genoa, Genova, Italy; 8 School of Engineering and Management Vaud (HEIG-VD), HES-SO University of Applied Sciences and Arts Western Switzerland, Yverdon-les-Bains, Switzerland; 9 Department of Diagnostic, Interventional and Pediatric Radiology (DIPR), Inselspital, Bern University Hospital, University of Bern, Bern, Switzerland; 10 Translation Imaging Center (TIC), Swiss Institute for Translational and Entrepreneurial Medicine, Bern, Switzerland

**Keywords:** Health Sciences, Neuroscience, Biotechnology and bioengineering

## Abstract

Magnetic resonance imaging (MRI) is essential for examining ocular pathologies, but conventional protocols are prone to motion artifacts caused by eye movements. Here, we present a protocol for integrating eye tracking (ET) with MRI to achieve high-resolution, motion-robust imaging of the eye. We describe acquisition, reconstruction, and post-processing steps combining ET and MRI data. This approach improves structural detail, enhances image quality, and strengthens MRI’s diagnostic value in ophthalmology while paving the way for replacing hardware-based ET with machine learning solutions.

For complete details on the use and execution of this protocol, please refer to Franceschiello et al.^1^

## Highlights

• Steps for synchronizing the eye-tracking system with MRI to achieve motion-robust images
• Instructions for running the visual fixation task during a radial T1w-LIBRE acquisition
• Procedures for binning k-space readouts by gaze into motion-resolved reconstructions
• Guidance on validating image quality against eye-tracker angles and ocular biometry

## Abstract (editor highlights)

Publisher’s note: Undertaking any experimental protocol requires adherence to local institutional guidelines for laboratory safety and ethics.

## Graphical abstract

**Figure:**
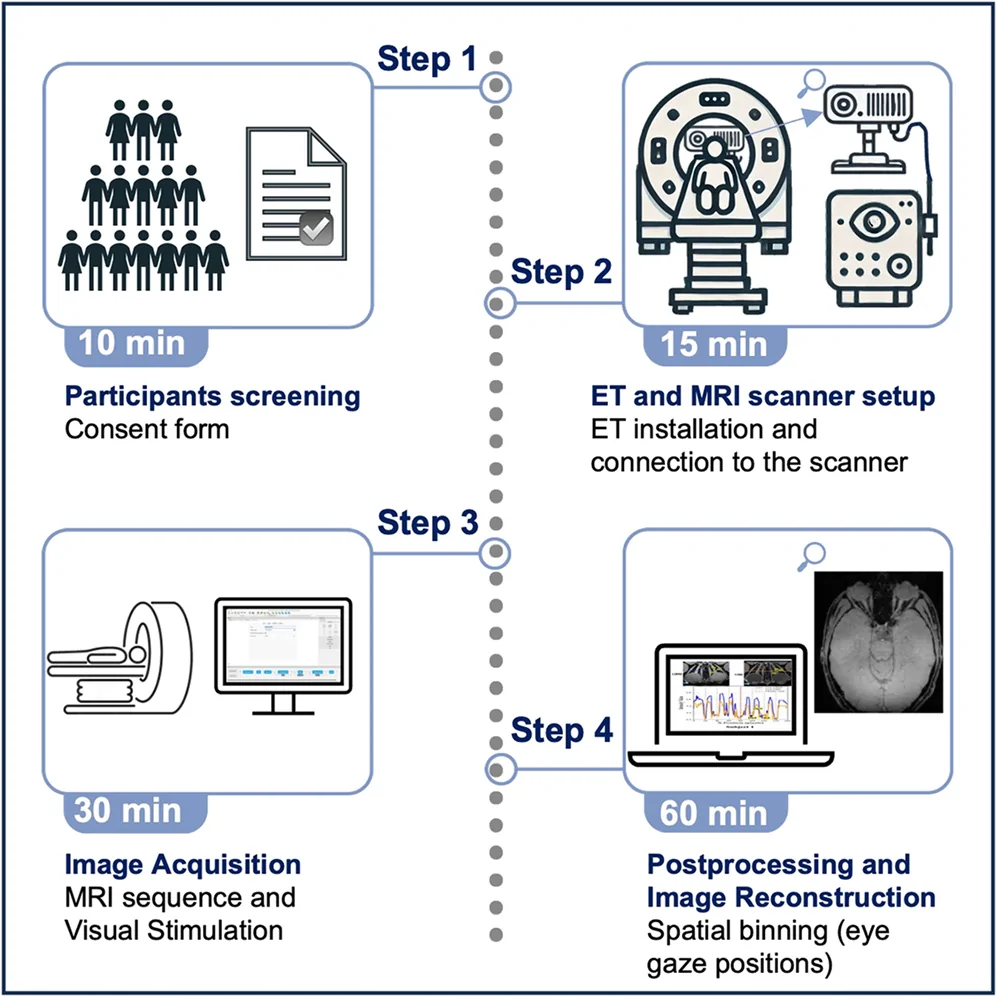

## Before you begin

Ocular pathologies—including tumors, vascular malformations, and inflammatory or degenerative conditions of the eye and orbit—often require detailed anatomical characterization beyond standard ophthalmological exams.^2^^,^^3^^,^^4^

MRI offers excellent soft-tissue contrast without ionizing radiation, but eye movements during scanning introduce motion artifacts that degrade resolution and can obscure pathological findings—a persistent challenge for MR ophthalmology (MR-Eye).^5^^,^^6^^,^^7^^,^^8^^,^^9^

This protocol describes the specific steps for setting up the system to acquire MR images of the eyes, with synchronized eye-tracking trajectories. This setup ensures proper data collection for motion-resolved compressed-sensing (CS) reconstruction of the eyes.

### Innovation

Conventional ocular MRI treats eye motion as an artifact to be suppressed: participants are asked to fixate, and data corrupted by residual movement are discarded or averaged. This protocol inverts that logic and makes eye motion the organizing variable of the reconstruction. Gaze is recorded continuously with an MR-compatible eye tracker whose trajectories are hardware-synchronized to the scanner, and every k-space readout of a 3D radial phyllotaxis T1w-LIBRE acquisition is assigned retrospectively to the gaze position held while it was sampled. A motion-resolved compressed-sensing reconstruction then recovers a separate high-resolution volume for each eye position, exploiting the information shared between neighboring gaze states instead of rejecting it.

Three features distinguish this workflow from existing MR-Eye approaches. First, it removes the fixation requirement that limits ocular MRI in patients unable to hold gaze, while retaining millimeter-scale resolution on a standard clinical 3T system. Second, it pairs a water-excitation LIBRE sequence, which suppresses orbital fat with low sensitivity to B0 inhomogeneity, with a non-Cartesian trajectory whose uniform k-space coverage survives arbitrary binning of the readouts. Third, it is reproducible end to end: the acquisition exists in vendor-native (Siemens IDEA) and vendor-agnostic (Pulseq) implementations, the reconstruction is distributed in the open-source Monalisa toolbox, and step-by-step standard operating procedures are published online.

Together, these choices turn an imaging confound into the signal that drives reconstruction and make dynamic ocular MRI deployable beyond the laboratory where it was developed.

### Protocol versions: DEBI, MR-Eye Track, and MR-Eye 2.0

Table 1 summarizes how the original DEBI protocol relates to its two derivatives. DEBI is the foundational implementation; MR-Eye Track emphasizes open, vendor-agnostic acquisition and reconstruction, and MR-Eye 2.0 adds multi-contrast imaging.

**Table 1:** Comparison of the DEBI protocol and its derivatives (MR-Eye Track, MR-Eye 2.0)

Feature	DEBI (original)	MR-eye track	MR-eye 2.0
Reference/implementation	Franceschiello et al., 2020 (foundational study)	HEIG-VD collaboration; open, vendor-agnostic	SNF-funded extension; multi-contrast; https://osf.io/fw6ut
Visual paradigm	4×4 grid, 16 positions × 6 repetitions × 5 s	4 positions × 30 repetitions × 5 s + 2 × 30 s central fixation	grid-based fixation task
Fixation target	Grey dot for central fixation and moving target on grid	Two circles and a cross for central fixation and moving target on grid	Dot changing color for central fixation
Approx. task duration	≈8 min	≈11 min	≈40 min
Head coil	20-channel head-neck coil	64-channel head-neck coil	64-channel head-neck coil
MRI sequence(s)	T1w-LIBRE	T1w-LIBRE	T1w-LIBRE, T1w-VIBE, T2-prep-LIBRE, T2w-TSE
Sequence framework	vendor-native Siemens IDEA	open-source Pulseq (vendor-agnostic)	open-source Pulseq (vendor-agnostic)
Reconstruction	in-house CHUV k–t sparse SENSE	open-source Monalisa toolbox	open-source Monalisa toolbox
Eye tracked	right eye (monocular)	right eye (monocular)	right eye (monocular)
Infrared mirror	mounted on head coil each session	pre-attached to head coil	pre-attached to head coil
Data/code	available on request	GitHub (MR-EyeTrack); Zenodo (planned); Monalisa	Github (2.0 MR-Eye); Zenodo (planned); Monalisa

### Institutional permissions

All scientific research on human participants must be approved by a local ethical committee before beginning. The study protocol in Franceschiello et al,^1^ was approved by the cantonal ethics committee (protocol number 2018–00240). A novel version of the protocol (MR-Eye Track) was submitted to the Swiss BASEC (Business Administration System for Ethics Committees) and received positive approval from the corresponding local Ethics Committee on June 27, 2024 (protocol number 2024–00165). We have obtained permission from the copyright holder (Publication: Progress in Neurobiology; Publisher: Elsevier) to reuse Figures 1, 3, and 4 from the original manuscript; the corresponding permission document is uploaded to the submission as a supplemental file set. These correspond to Figures 3, 5, and 6 of the present protocol.

**Figure 1:**
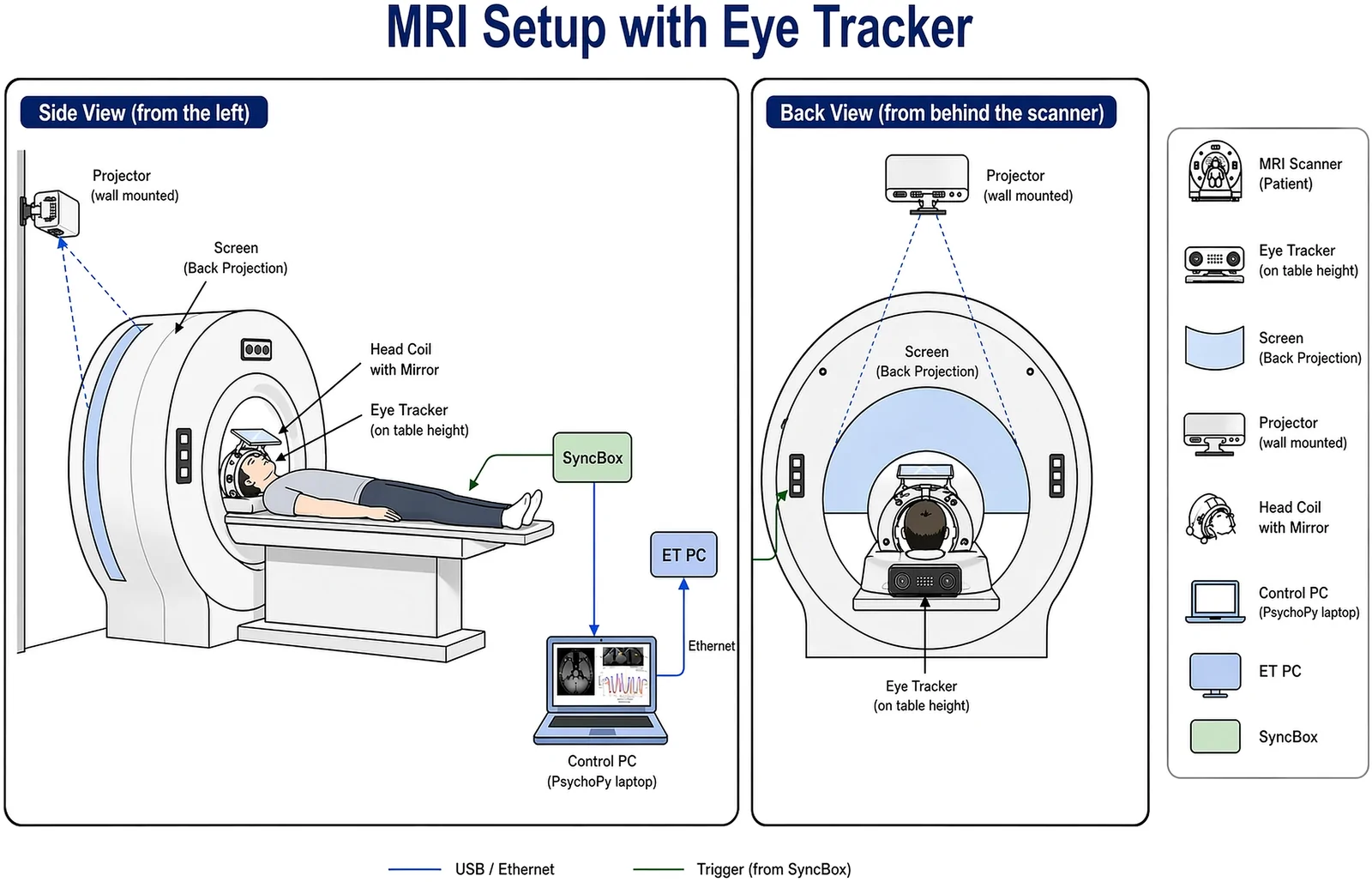
Overall experimental setting Schematic of the main hardware connections inside and outside the MRI scanning room, including the eye-tracker, stimulus computer, SyncBox, and scanner interface. The SyncBox, ET PC and PsychoPy laptop are outside the scanner room.

### Preparing the experimental materials

**Timing: 2–5 days (preparatory work, performed once before data collection)**

1. Implement a visual stimulation paradigm to guide controlled eye movements during MRI acquisition, enabling retrospective motion-resolved image reconstruction based on eye position.
  ***Note:*** The stimulation can be programmed in *PsychoPy*^10^ and should display a fixation target through the mirror mounted on the head coil.
  a. Move the fixation target sequentially across a predefined 4 × 4 grid of visual positions, holding each location for 5 s.
  b. Repeat all 16 positions 6 times in randomized order.
  c. Between target displacements, have participants fixate on a central target that serves as the starting and ending position of each eye movement.
  d. Synchronize stimulus presentation with the MRI acquisition using a synchronization box (SyncBox S, NordicNeuroLab) configured to send an ASCII “s” character that triggers both simultaneously.
  e. Configure the *PsychoPy* script to wait for receipt of the “s” character before initiating the stimulation sequence.
    ***Note:*** The total stimulus duration is therefore 480 s (16 positions × 6 repetitions × 5 s = 8 min).
    **CRITICAL:** Precise synchronization between MRI acquisition, stimulus presentation and eye-tracking recordings is required, as temporal alignment is necessary for the subsequent motion-resolved compressed-sensing reconstruction.
    ***Note:*** In the updated versions of the protocol (MR-Eye Track and 2.0 MR-Eye), the fixation stimuli are composed of two circles and a cross, a fixation shape designed to reduce undesired eye movements.^11^ For MR-Eye Track, the total stimulus duration is 4 positions × 30 times × 5 s + 30 s × 2 times = 660 s = 11 min, approximately.
    ***Note:*** The visual experiment must include ET calibration and validation phase before the visual task.
    ***Note:*** Right-eye movements are tracked using the ET system synchronized with the MRI scanner.
    ***Note:*** The visual stimulation task should have the same duration as the MRI sequence.
2. Prepare the MRI sequence to run along with the visual stimulation.

***Note:*** For our case, we opted for a water excitation technique, T1w LIBRE (Lipid Insensitive Binomial Off-Resonant Excitation) GRE with 3D radial spiral phyllotaxis trajectory,^12^ in the vendor-native environment (Siemens’ IDEA). This sequence provides robust fat signal suppression with reduced B_0_ inhomogeneity. For the newer version of the protocol, we developed an equivalent version with the open-source framework Pulseq,^13^ making it vendor agnostic.

3. Choose a reconstruction framework.

***Note:*** For our case, we chose an in-house reconstruction framework (CHUV), using a k–t sparse SENSE algorithm.^14^ For the newer version of the protocol, we used our open-source Monalisa reconstruction framework.^15^

***Note:* Acceptable alternatives.** The essential components of this protocol are (i) an MR-compatible eye-tracking system with an infrared mirror on the head coil, providing gaze trajectories synchronized to the MRI acquisition; (ii) a hardware trigger/synchronization device linking stimulus presentation, eye tracking and the scanner; (iii) an anatomical sequence with a non-Cartesian (here 3D radial phyllotaxis) trajectory and robust fat suppression; and (iv) a motion-resolved compressed-sensing reconstruction. The following are substitutable: the eye-tracker model (we used the EyeLink 1000 Plus, but any MR-compatible tracker delivering time-stamped gaze data can be used), the stimulus software (PsychoPy), the synchronization box (any device delivering scanner-locked triggers), the sequence-programming framework (vendor-native Siemens IDEA or the vendor-agnostic open-source Pulseq implementation) and the reconstruction framework (in-house CHUV k–t sparse SENSE, the open-source Monalisa toolbox, or any other reconstruction toolbox). The scanner vendor itself is not essential when the sequence is implemented in Pulseq.

4. Recruit participants.
  a. Distribute flyers, both physically and electronically (e.g., via email lists).
  b. Log each interested candidate in the internal coded spreadsheet.
  c. Record an email address for each candidate for sending the pre-study documents.

### Participant screening

**Timing: 10 min**

5. Have participants complete the pre-study forms (5 min).
  a. Obtain written informed consent, ensuring that participants are aware of the content of the study and any associated risks or benefits.
  b. Have participants complete the MR safety form to check their compatibility with MR scanning.
  c. Have participants complete the general data-reuse consent form.
  d. Have participants complete the reimbursement form.
6. Have participants complete a demographic form (5 min).

***Note:*** The demographic form includes age, education level, sex assigned at birth, history of neurological and/or psychiatric diseases, medication, etc.

**CRITICAL:** It is strongly recommended to conduct a pilot study before the experiment and run a power analysis with these data to estimate the optimal number of participants based on the expected effect-size.

### Preparation outside the scanner room

**Timing: 5–10 min**

***Note:*** See Figure 1 for the overall experimental setting.

***Note:*** If it is the first time connecting the stimulus computer to the eye tracker (ET), the IP address needs to be configured. For the next time, it will already be configured properly (Step 7).

***Note:*** Photographs of the physical setup and full session-preparation instructions are available at https://mattechlab.github.io/sops/debi/data-collection/1_session_prep/.

7. Configure the IP address on the stimulus computer, also referred to as the *PsychoPy* laptop in Figure 1, equipped with Windows OS.
  a. Connect the ET computer to the stimulus computer via the Ethernet cable.
  b. Go to Control Panel -> Network and Internet -> Network Connections.
  c. Double-click on the Ethernet connection.
  d. Select Internet Protocol Version 4 (TCP/IPv4) and click Properties.
  e. Update the IP address to 100.1.1.2 and the subnet mask 255.255.255.0.
8. Turn on the projector next to the MRI scanner room.
  a. Check that the projector beam is directed into the scanner room.
9. Set up the stimulus computer on the control room’s desk.
  a. Insert the HDMI into the *PsychoPy* laptop to monitor the visual stimuli on the screen (HP 24mh).
    ***Note:*** The HDMI should be from the 3-cable bundle (which connects the ET PC, the projector and the PsychoPy laptop).
  b. If the monitor does not automatically switch the screen source, use the switch button to manually change it.
  c. Change the laptop screen resolution to 800 × 600 pixels.
    ***Note:*** This resolution ensures that the whole screen is projected to the participant inside the scanner.
  d. Ensure that the PC beneath the monitor remains turned on.
  e. Connect the USB cable from the SyncBox to the laptop.
  f. Plug the Ethernet from the ET computer to the *PsychoPy* laptop.
10. Set up the SyncBox S by NordicNeuroLab.
  a. Plug the RJ45-coaxial cable into the SyncBox.
  b. Route the cable through the interface in the scanner room wall and connect it to the scanner.
    ***Note:*** The port position must match on both sides of the interface: if the cable enters position 3 on the outside, it must exit position 3 on the inside.
  c. Turn on the SyncBox.
  d. Check synchronization.
    i. Enter the Synchronization mode by selecting it and pushing the enter button.
    ii. Hit the down arrow button until you find *Send trigger pulses* to PC.
    iii. Push the enter button every time you want to send an “s” character.
    iv. Check that the *PsychoPy* laptop types those triggers (e.g., on an open editor receiving keypresses, or the shell prompt, or looking at your open log).
  e. Start a new SyncBox *Simulation* session:
    i. Push the up-arrow button until you find *Start Session.*
    ii. Push the enter button to start the session.
      ***Note:*** The SyncBox will now wait for the scanner's trigger signal to forward it.
    iii. Configure the TR to *2500 ms*, according to the sequence.
      ***Note:*** The TR determines the interval between two triggers; it is only used to flag the triggers.
      **CRITICAL:** Synchronization between the SyncBox, the scanner and the eye tracker is a critical step. Before every session, verify that the “s” trigger is received by the stimulus laptop from the Syncbox signal; even a few-millisecond offset propagates into incorrect binning of the MRI readouts. Re-check synchronization after any scanner software upgrade.
11. Set up the Eye-Tracker.
  a. Prepare the ET device.
    i. Exit the Scanning Room and fetch the ET arm.
    ii. Unscrew and remove any lens currently mounted on the ET.
    iii. Store the removed lens in its plastic bag inside the lens box.
    iv. Carefully install the 50 mm MR-compatible lens onto the ET.
      ***Note:*** The default focus for minimum distance is typically set to around 1 m. However, the exact value can vary depending on the specific participant and set up each time. It is recommended to start with the focus at 1 m and adjust from there for optimal focus during setup.
  b. Prepare the ET system.
    i. Make sure the ET’s PC is plugged in.
    ii. Before turning on the ET computer, ensure it is connected to the *PsychoPy* laptop via Ethernet cable.
    iii. Switch on the ET’s PC using the power-on button at the front.
    iv. Select “Eyelink” when given the option of which operating system to launch.
    v. Initialize the ET software from the ET workstation here by typing “*elcl.exe*” in the terminal.
      ***Note:*** The command ‘*elcl.exe’* will only work and start the software once the eye-tracker device is connected to the ET PC. If you encounter any error messages, please ensure the eye-tracker is connected to the PC inside the scanner room and try running the command again.
  c. Place the infrared-mirror onto the head coil.
    i. Detach the standard mirror frame from the head coil, if it is placed there.
    ii. Take the protective box containing the infrared mirror.
      ***Note:*** Always keep the infrared mirror protected by its mask unless it is in use.
    iii. Put on new latex gloves.
    iv. Extract the infrared mirror from its box and carefully attach it to the head coil.
      ***Note:* Description of the custom infrared-mirror assembly.** The infrared-mirror assembly is a custom component and is described here to facilitate replication. It consists of a front-surface mirror that reflects near-infrared light, held in a lightweight, MR-compatible (non-ferromagnetic) frame that clips onto the same rails on the head coil normally used for the standard visual-feedback mirror. When mounted it sits above the participant’s right eye and folds the infrared optical path so that the eye-tracker camera and infrared illuminator, positioned at the rear of the bore, can image the pupil. Because it is a front-surface mirror it is the most delicate part of the setup: it cannot be resurfaced, so it is always handled with gloves and kept covered by a protective mask when not in use. A photograph of the mirror is available at: https://mattechlab.github.io/sops/assets/debi_protocol/selected/22_anno_head_coil_mirror.png.
      ***Note:*** In the new version of the protocol, MR-Eye Track protocol, the infrared mirror is already attached to the head coil.

### Preparation inside the scanner room

**Timing: 5–10 min**

12. Place the necessary equipment.
  a. Bring the plexiglass panel from the technician’s room into the scanning room to support the ET arm.
    i. Place the panel at the end of the scanner’s bore, with the top side (indicated by a sign) facing upwards and the plastic feet facing down.
    ii. Align the edge of the plexiglass with the edge of the illuminated MRI rails to ensure repeatable positioning of the ET.
      ***Note:*** The plastic feet prevent the panel from sliding. Scotch-tape references on the plexiglass mark the ET arm position, ensuring positioning repeatability across participants and experiments.
  b. Enter the scanning room and place the ET arm on top of the plexiglass panel with the two posterior feet of the ET arm aligned within the two corner markers made of scotch tape.
    ***Note:*** Hold the ET arm firmly, because the magnetic field imposes some resistance.
  c. Unroll and connect the cables of the ET with the ET’s PC through the wall connectors inside the scanning room.
  d. Take the half-circle one-direction screen from the table behind the scanner.
  e. Place the screen onto the glass plate, between the projector and the eye tracker, so that it reflects the projector’s image.
  f. Plug the EXT trigger cable into the scanner.
  g. Check that the link is properly established by typing *ping 100.1.1.1* in a terminal.
    ***Note:*** The ET should respond to the echo requests sent from the terminal.
13. Place the participant.
  a. Place the subject on the bed.
    i. Provide the participant with a blanket, earplugs, sandbags, leg, and head cushions.
    ii. Adjust the head coil and the mirror with the infrared plate.
    iii. If necessary, apply pads to adjust the participant’s head position so that the forehead is tightly positioned against the head coil.
      **CRITICAL:** Head positioning is a critical step. The forehead must be firmly against the head coil, using pads if needed, both to minimize head motion and to keep the eye within the eye-tracker’s field of view for the whole session.
    iv. Ensure the participant does not cross their legs or arms.
    v. Provide the participant with the emergency button and explain that it can be pressed in case of an emergency.
  b. Adjust the bore’s position.
    i. Twist the knob to adjust the height of the bed and wait for it to stop.
    ii. Gently move the participant inside the bore with the manual controls.
    iii. Instruct the participant to close their eyes.
    iv. Turn on the red light to localize the head.
    v. Place one hand on the head coil, then turn the knob left or right to align the red light with the mark on the head coil.
    vi. Once aligned, turn off the red light and instruct the participant to open their eyes.
    vii. Turn off the ventilation and set the scanner light to the minimum level, to prevent blinking or drying eyes.
    viii. Press the central button of the scanner to move the participant table inside the scanner bore.
  c. Adjust the lens of the eye-tracker.
    i. Go to the back of the scanner.
    ii. Point the lens of the eye-tracker towards the participant’s right eye.
    iii. Set the screw on the lens to its default position (around 1) for convenient adjustment.
    iv. Rotate the lens until the pupil is in focus on the screen in camera mode.
    v. Fine-tune the focus until the image is sharp, with both the pupil and eyelashes well defined.
      **CRITICAL:** Correct focusing of the eye-tracker lens is a critical step. The pupil must be sharply in focus with the eyelashes clearly defined; poor focus prevents reliable pupil/CR detection and compromises the gaze-based binning.

### ET calibration

**Timing: 10–15 min**

***Note:*** Check the ET calibration section in the SOPs website for detailed information: https://mattechlab.github.io/sops/debi/data-collection/1_session_prep/#et-calibration and https://mattechlab.github.io/sops/general/et-related/notes-et/.

***Note:***Figure 2 shows the eye-tracker camera setup, calibration and validation procedure.

14. Brief the participant before leaving the scanner room.
  a. Inform the participant that you are leaving the room and will shortly return for the final preparation.
  b. Instruct the participant to follow the moving dot with their eyes during the task, without anticipating its next position.

**Figure 2:**
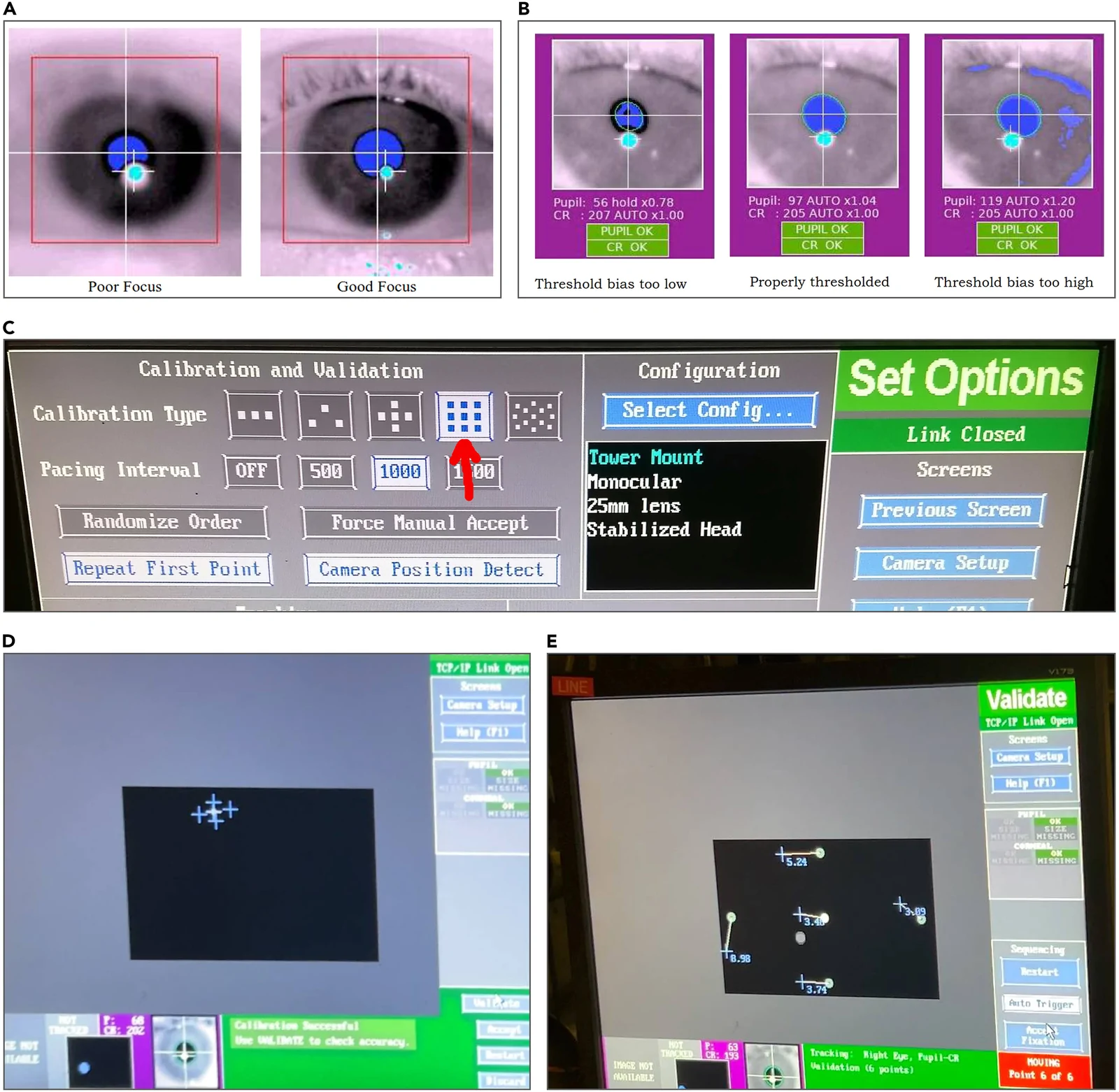
Eye-tracker camera setup, calibration and validation procedure Successive stages of the eye-tracking setup as displayed on the EyeLink Host PC. (A) Camera focus adjustment. (B) Pupil threshold adjustment, showing under-, correct and over-thresholding. (C) Configuration screen: tower mount, monocular, 25 mm lens, stabilized head, with the nine-point calibration grid selected (red arrow). (D) Successful calibration. (E) Validation, reporting the angular deviation at each target. Please note that some of the images used in this figure are sourced from the EyeLink 1000 Plus User Manual. These panels illustrate the EyeLink Host PC interface as shipped; the configuration used in this protocol is a 50 mm MR-compatible lens with 5-point calibration.

***Note:*** The target locations are randomized, so the participant cannot predict the next position.

15. Open *PsychoPy* on the *PsychoPy* laptop.

***Note:*** If you are connecting the eye tracker to the PsychoPy laptop for the first time, configure the IP address first (step 7).

16. Run the experiment from the terminal of the *PsychoPy* laptop by typing *python experiment.py.*

***Note:*** All experiments must include a calibration and validation phase before the experiment itself. These phases can also be configured and launched from the ET PC console.

17. Run the calibration.
  a. Once the stimulation begins, follow the on-screen messages to run the calibration and check that the following options are selected:
    i. Calibration Type: 5 points calibration.
    ii. Sampling rate: 1kHz.
    iii. Tracking mode: Pupil-CR.
    iv. Pupil Tracking: Ellipse.
    v. Camera Position: Right.
18. Apply threshold to find the pupil.
  a. On the ET PC, click *Apply Threshold.*
  b. Check that the pupil is detected, i.e., that a blue cross appears on the eye.
    ***Note:*** If you encounter issues, check the lighting inside the scanner (ensure it is not too bright or too dim), verify the participant's head position inside the coil and adjust the mirror and half-circle screen positioning if needed to improve the pupil’s detection.
  c. Once the calibration starts, accept the calibration points when they turn green by clicking *Accept Fixation.*
    ***Note:*** If the calibration points form a cross, it is the perfect calibration.
  d. Check the stability of the accepted points and the overall calibration score before moving to the next step.
    ***Note:*** A successful calibration is indicated by the message “calibration successful” in green at the bottom of the screen.
    **CRITICAL:** Calibration quality is a critical step. Proceed only if the calibration is accepted with a stable, low-error score (ideally the accepted points form a regular cross when using 5-point calibration); a poor calibration cannot be corrected retrospectively and degrades the motion-resolved reconstruction.
19. Follow up with the validation.
  ***Note:*** In an ideal situation, you should see the reference dot in the center of the screen and a second calibration dot that corresponds to the pupil calibration. The calibration dot should be stable, with only small movements around the reference dot.
  a. If the calibration dot is unstable and moves far from the reference dot, click *Restart*, adjust the pupil threshold and redo the calibration.
  b. Once the calibration dot is stable, click *Accept Fixation* to proceed with the validation.
20. Prepare the participant for the start of the scan.
  a. Enter the scanner room and inform the participant that you are leaving the room and will now close the door to start.
  b. Tell the participant that you will speak to them shortly through the speaker to check that communication is working.
21. Exit the scanning room and close the scanning room door.

## Key resources table

**Table:** 

REAGENT or RESOURCE	SOURCE	IDENTIFIER
**Deposited data**		

MR-Eye Track data	Derivative of this study	Zenodo (DOI to be assigned at publication)
MR-Eye 2.0 data	Derivative of this study	Zenodo (DOI to be assigned at publication)
Python code to run post-processing of eye tracking data used for generating binning mask for motion-resolved reconstruction	Derivative of this study	https://mattechlab.github.io/sops/debi/data-processing/1_2_et_QAQC_of_mreye_tracking/
MATLAB code to run motion-resolved reconstruction based on open-source toolkit Monalisa.	Derivative of this study	https://doi.org/10.1101/2025.06.06.658299
*PsychoPy* experiment program for displaying visual stimuli	This study (version for MR-Eye Track in the link)	https://github.com/MattechLab/MR-EyeTrack/blob/dev/visual_stimuli/mreyetrack_16points.py
Additional standard operating procedures (SOPs), including step-by-step data processing guides for the DEBI protocol and its derivatives	Derivative of this study	https://mattechlab.github.io/sops/

**Experimental models: Organisms/strains**		

Human participants (Homo sapiens), healthy adult volunteers, both sexes (4 males, 5 females), aged [27–34] years (N = 9); no genotype selection	This study	N/A

**Software and algorithms**		

Python version 3.8	Python Software Foundation	https://www.python.org
Monalisa Toolkit	https://github.com/MattechLab/monalisa	https://mattechlab.github.io/monalisa/
MATLAB 2024b	The MathWorks	https://ch.mathworks.com/fr/products/matlab.html
PsychoPy 24.2	PsychoPy 24.2	https://www.psychopy.org/
SPM12	Functional Imaging Laboratory, UCL Queen Square Institute of Neurology, London, UK	https://www.fil.ion.ucl.ac.uk/spm/
Pulseq	V1.5	https://pulseq.github.io/
EyeLink Developers Kit/EDF2ASC converter (EDF to BIDS conversion)	SR Research	https://www.sr-research.com/support/

**Other**		

3T clinical scanner MAGNETOM PrismaFit	Siemens Healthineers	https://www.siemens-healthineers.com/magnetic-resonance-imaging/3t-mri-scanner/magnetom-prisma
SyncBox S	NordicNeuroLab	https://www.nordicneurolab.com/products/fmri-acquisition
EyeLink 1000Plus	SR Research	https://www.sr-research.com/eyelink-1000-plus/
64-channel head-neck coil	Siemens Healthineers	https://www.siemens-healthineers.com/magnetic-resonance-imaging/options-and-upgrades/coils/64-channel-head-neck-coil
Infrared mirror	Custom	N/A
Projector	Sony std.	N/A
Scanner’s half-circle screen	N/A	N/A
50 mm MR-compatible lens for EyeLink 1000 Plus	SR Research	https://www.sr-research.com/eyelink-1000-plus/
20-channel head-neck coil	Siemens Healthineers	https://www.siemens-healthineers.com/magnetic-resonance-imaging/options-and-upgrades/coils
IOLMaster 700 optical biometer	Carl Zeiss Meditec	https://www.zeiss.com/meditec/en/products/iolmaster-700.html
HP 24mh monitor (stimulus preview screen)	HP	https://www.hp.com/
Plexiglass support panel for the eye-tracker arm	Custom	N/A

## Step-by-step method details

In this section, we present the end-to-end pipeline of the protocol, from image acquisition to data processing. Figure 3 shows the acquisition-reconstruction pipeline.

**Figure 3:**
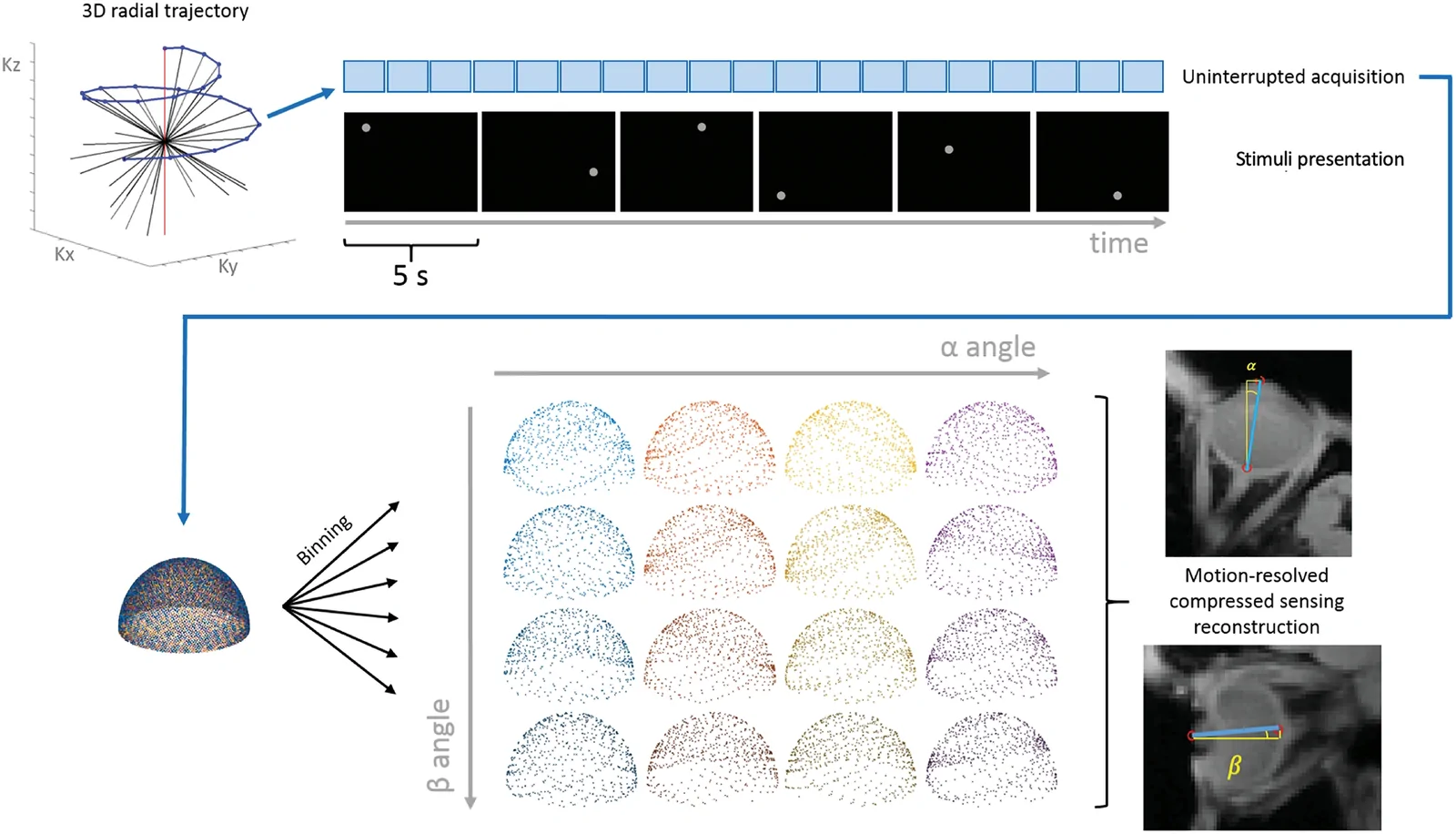
A schematic illustration of the framework from image acquisition to reconstruction Each one of the 16 final motion-resolved images (associated to the locus of stimulus presentation) is obtained from an under-sampled set of about 5000 k-space readouts (i.e., 81,906/16). Nevertheless, uniform k-space coverage regardless of the stimulus presentation order is ensured by the radial phyllotaxis trajectory properties. The stimulus presentation scheme was parametrized by the angles (α, β). The idea at the basis of the k-t sparse SENSE algorithm is that close-by positions (such as the blue and the red cloud of dots) share a high amount of information about the k-space, allowing to perform image reconstruction from an under-sampled acquisition of k-space lines. Reproduced from Franceschiello et al.^1^ with permission from Elsevier (Progress in Neurobiology).

### Image acquisition

**Timing: 15–20 min**

In this phase, participants perform the visual stimulation task during the MRI scan. The ET recordings are stored in the *PyschoPy* laptop, and the raw data of the anatomical acquisition on the scanner PC. The purpose of the imaging session is to distinguish between gaze phases (eye movement, including saccades, blinks, and fixations) for posterior analysis.

***Note:*** In our study, we used a 3T clinical MRI scanner (MAGNETOM Prisma fit, Siemens Healthineers, Forchheim, Germany) through a 3D radial-spiral phyllotaxis trajectory using a 20-channel head coil to obtain the MR images.

1. Before starting the experiment, check that the PC running the visual stimulation task is connected to the screen system of the scanner.
2. Check the ET setup and calibration.
3. Run the *PsychoPy* experiment and the scanning protocol.
  a. Wait for the sentence regarding the initial description of the task: “In this task you will see…”
  b. On the IDEA UI, activate the EXT trigger to start both the stimulation and the ET recording.

***Note:*** The protocol includes a head scout (20-ch and 64-ch head coils for this study and for the derivative studies MR-Eye Track and 2.0 MR-Eye, respectively), a high-resolution anatomical image (MPRAGE) and the main sequence, T1w LIBRE.

***Note:*** At the end of the ET calibration, we are ready to continue the experiment.

**CRITICAL:** Trigger configuration is a critical step. Confirm that the EXT trigger is enabled in the IDEA Physio section and that a single trigger simultaneously starts the MRI acquisition and the eye-tracking recording; verify on the ET PC that the sample counter is increasing.

4. Session Completed.
  a. At the end of the stimulation, press the “t” key on the experimental laptop.
  b. Press the round button on the SyncBox to stop the running session.
  c. Inform the participant that the session has concluded.

***Note:*** You can proceed with the tear-down protocol.

### Session teardown

**Timing: 10 min**

5. Showing the Participant out.
  a. Enter the scanner room and announce yourself to the participant saying that you will get them out in a few seconds.
  b. Extract the participant by pressing the extraction button and then gently rolling the central knob.
    ***Note:*** Alternatively, press the Home button.
  c. Remove the upper side of the head coil:
    i. Unplug the head coil from the bed connector.
    ii. Lift the lever that releases the upper part of the coil and put it aside (e.g., inside the bore or on a chair next to the scanner).
  d. Help the participant sit down.
  e. Help the participant step down and accompany them out to the control room.
  f. Help the participant recover their personal belongings and direct them to where they may change clothes if necessary.
  g. Ask the participant to sign the receipt form for compensation.
    ***Note:*** The signed form is then processed by the finance department at the school.
6. ET setting.
  a. Place the half-circle screen back to the table behind the scanner.
  b. Unplug the two cables (signal and power) connected to the ET arm.
  c. Roll the two ET cables and put them in the cupboard inside the scanning room.
  d. Remove the mirror frame from its rails mounted on the head coil and lay it on the bed.
  e. Put the gloves on and cover the infrared mirror with a mask for storage.
7. Clearing up the Scanner.
  a. Unplug the cable on the scanner from the SyncBox and roll it back to the shelf.
  b. Remove the used blankets and bedsheets one by one.
  c. Unfurl each item to let out any forgotten objects before folding it.
  d. Dispose of each item in the appropriate bin (soiled-linen bag for fabric, trash for disposables).
  e. Dispose of all single-use sanitary protections (padding covers, earplugs, etc.).
  f. Put the pillows back in their designated storage places.
  g. Remove the head coil and put it in the scanner’s bore.
  h. Remove the back padding elements and put them back in their designated storage.
  i. Reinstall the spine coil.
  j. Wipe the bed and the head coil (bottom and upper parts).
  k. Lock the head coil back with its bottom part without plugging the connectors.
  l. Put the head coil away with the other head-coils on the shelf next to the scanner.
  m. Return the bed to its *home* position by pressing the home button on the scanner (more info).
  n. Take the ET arm, the infrared mirror, and the plexiglass panel outside to the control room and store them in the ET/fMRI box.
  o. Exit and close the external door.

***Note:*** Everything that is removed for the experiment needs to be put back in place at the end of the experiment, i.e., position of the bed, head coil, emergency button, ears cover.

8. Collect ET data.
  a. Copy data from the subfolder of *PsychoPy* program into the hard drive.

***Note:*** The subfolder should include the files for one session where the.EDF files are the eye tracking records, and the.csv and.log files save the task messages and the corresponding timestamps.

9. Collect MRI raw data.
10. Export Twix raw data.
  a. Insert username and password.
  b. Press Ctrl+Esc to open the IDE terminal.
  c. In the IDE terminal: ideacmdtool -> type 4 -> type 6.
  d. Type Twix to open the Twix data browser.
  e. Select the data you want to copy.
  f. Right-click and select Copy Total RAID file.
  g. Select the destination (your hard drive).
  h. Make sure the flag on External Signal is turned on.
11. Export the DICOM data (directly reconstructed images from the scanner).
  a. Login as Superuser to enter the advance mode.
  b. Select the patient.
  c. Go to Export//File System//Browse, and select HD.
  d. Select the “Enhanced” option (1 DICOM/volume) instead of Interoperability (1 DICOM/slice).

***Note:*** The default option is Interoperability, so it must be changed manually.

12. Cleaning up the Control Room.
  a. Plug the SyncBox and the VGA projector back in where they were.
    ***Note:*** Leave the connections exactly as you found them.
  b. Cover the eye-tracker lens with the lid.
  c. Unscrew the 50 mm lens from the eye tracker and place it back in the bag tagged “50mm Lens for MRI use”.
  d. Check that the infrared mirror is covered with a mask and that everything is stored safely in the ET/MRI box.
  e. Store the ET/MRI box back to the office.
  f. Switch off ET PC.
  g. Switch off the projector.

### Data quality control

**Timing: 30 min**

13. MRI data quality control.
  a. Verify, based on data size, that all required *k-space* samples of the radial trajectories have been successfully acquired.

***Note:*** Image quality cannot be flagged directly at the scanner, since no reconstruction is available yet.

***Note:*** Any partial or corrupted data sample will be flagged for reacquisition.

***Note:* Objective post-reconstruction quality metrics.** SNR and CNR are computed on T1-weighted LIBRE motion-resolved volumes using segmentation-derived ROIs (propagated from a 5-sample reference template by registration and manually verified against the reconstruction). SNR is the ROI signal mean divided by the standard deviation of a 3D background ROI placed anterior to the globes; because this background region includes residual streak/undersampling artifacts rather than pure air, the resulting values are relative image-quality indices specific to this normalization and background placement. All-muscle SNR is computed from the four rectus ROIs (left/right lateral and medial rectus). To characterize fat suppression, CNR between a structure and orbital fat is computed as their absolute mean difference normalized by the background SD. In our dataset: SNR ≈ 2.5 (globe), 3.5 (rectus muscles), 3.8 (optic nerve); CNR vs orbital fat ≈ 2.7 (optic nerve) and 2.5 (rectus muscles). Because these indices are relative, we recommend users establish their own baseline (e.g. against a fully sampled or VIBE reference acquired with identical ROIs and background) and flag runs that fall substantially below it for re-binning or reacquisition.

14. Eye-tracking data quality control.
  a. Convert the eye-tracking raw data from EDF format into BIDS format with the software provided by EyeLink.
    ***Note:*** This conversion also applies to the derivative studies MR-Eye Track and 2.0 MR-Eye.
  b. Visualize the ET trajectories and analyze the distribution of gaze points along each direction.
  c. Identify the center of gaze points and the deviation, checking for the alignment with the visual stimuli.
  d. Compute statistics such as the mean, median, and standard deviation for eye movement durations, including saccade, blink, and fixation durations.
    i. Compare these statistics across subjects using violin plots.
    ii. If one subject’s movement durations differ significantly from the others, halt the processing loop for this sample to save time and maintain data quality.
    iii. Schedule a new session to replace the excluded sample.

### Data processing

**Timing: 1–2 h**

15. Motion-resolved reconstruction (Figure 4 shows the overall workflow of the image reconstruction).
  a. Use the processed eye-tracker data to bin the time intervals of each motion state.
    i. Match the k-space readouts that correspond to the same stimulus presentation, so that they contribute to the same motion-resolved 3D image.
      ***Note:*** In other words, the eye-tracker trajectories are used to separate the k-space readouts into different volumes.
  b. Perform the motion-resolved 5D image reconstruction (x-y-z-α-β dimensions) using a k–t sparse SENSE algorithm (image undersampling 8.8%).
    i. Exploit sparsity along both the α and β directions.
      ***Note:*** α and β represent the eye angular rotations in the horizontal and vertical directions, respectively.
      ***Note:*** The values of α and β are deduced from the eye-tracker recordings and correspond to those determined from the reconstructed images by a trained optometrist, once normalized.
      ***Note:*** With this protocol, reconstruction required approximately 1 h per subject, though this time is dependent on the available IT infrastructure.
      ***Note:* Computational requirements and reproducibility.** Motion-resolved reconstruction is performed in MATLAB. In the original DEBI implementation, it used the in-house CHUV k–t sparse SENSE framework; in the newer versions it is performed with the open-source Monalisa toolbox, which supports the non-Cartesian 3D radial phyllotaxis trajectory of the T1w/T2w-LIBRE sequences (reconstruction call: Steva, a single-frame least-squares reconstruction regularized with the l1-norm of the spatial image gradient). The inputs are the Siemens raw k-space data (Twix “.dat” files) together with the eye-tracking–derived binning mask (“.mat”); the reconstructed motion-resolved volumes are written in MATLAB “.mat” format (convertible to NIfTI for downstream analysis). Because of the matrix size, typically 240×240×240, the reconstruction is memory-intensive and should be run on a workstation or HPC node with at least 256 GB of RAM; we recommend an HPC when reconstructing several subjects. Typical reconstruction time is approximately 1 h per subject, though this depends on the available IT infrastructure. The number of k-space readouts preserved after eye-motion binning was around 5,000 readouts. Full step-by-step instructions, dependencies and example scripts are provided in the Monalisa documentation (https://mattechlab.github.io/monalisa/) and the MRI data-processing SOP (https://mattechlab.github.io/sops/debi/data-processing/2_MRI_post_processing/).

**Figure 4:**
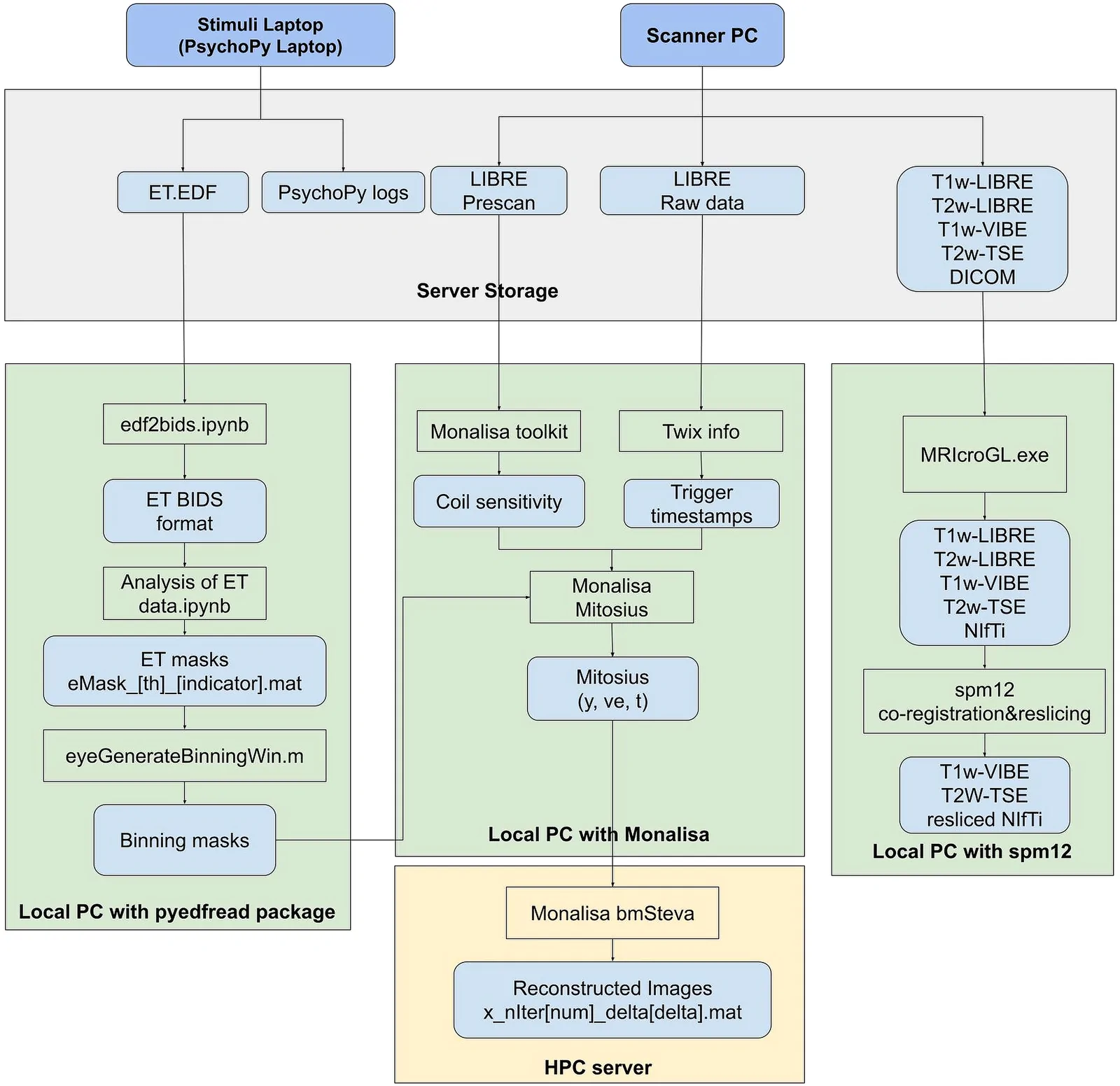
The overall workflow of the data processing after the acquisition

## Expected outcomes

We developed a novel MRI protocol for acquiring 3-dimensional images while the eye freely moves. Eye motion serves as the basis for image reconstruction, rather than an impediment. We fully reconstruct videos of the moving eye and head. We quantitatively validate data quality with millimeter resolution in two ways for individual participants. First, eye position-based reconstructed images correlated with simultaneous eye-tracking. Second, the reconstructed images preserve anatomical properties; the eye axial length measured from MR images matched that obtained with ocular biometry.^1^ The technique operates on a standard clinical setup, without specialized hardware, facilitating wide deployment. In clinical practice, we anticipate that this may help reduce burdens on both patients and infrastructure, by integrating multiple varieties of assessments into a single comprehensive session. More generally, our protocol is a harbinger for removing the need of fixation, thereby opening new opportunities for ethologically valid, naturalistic paradigms, the inclusion of populations typically unable to stably fixate, and increased translational research such as in awake animals whose eye movements constitute an accessible behavioral readout. Figure 5 shows the quality of the reconstructed images using motion-resolved eye binning.

**Figure 5:**
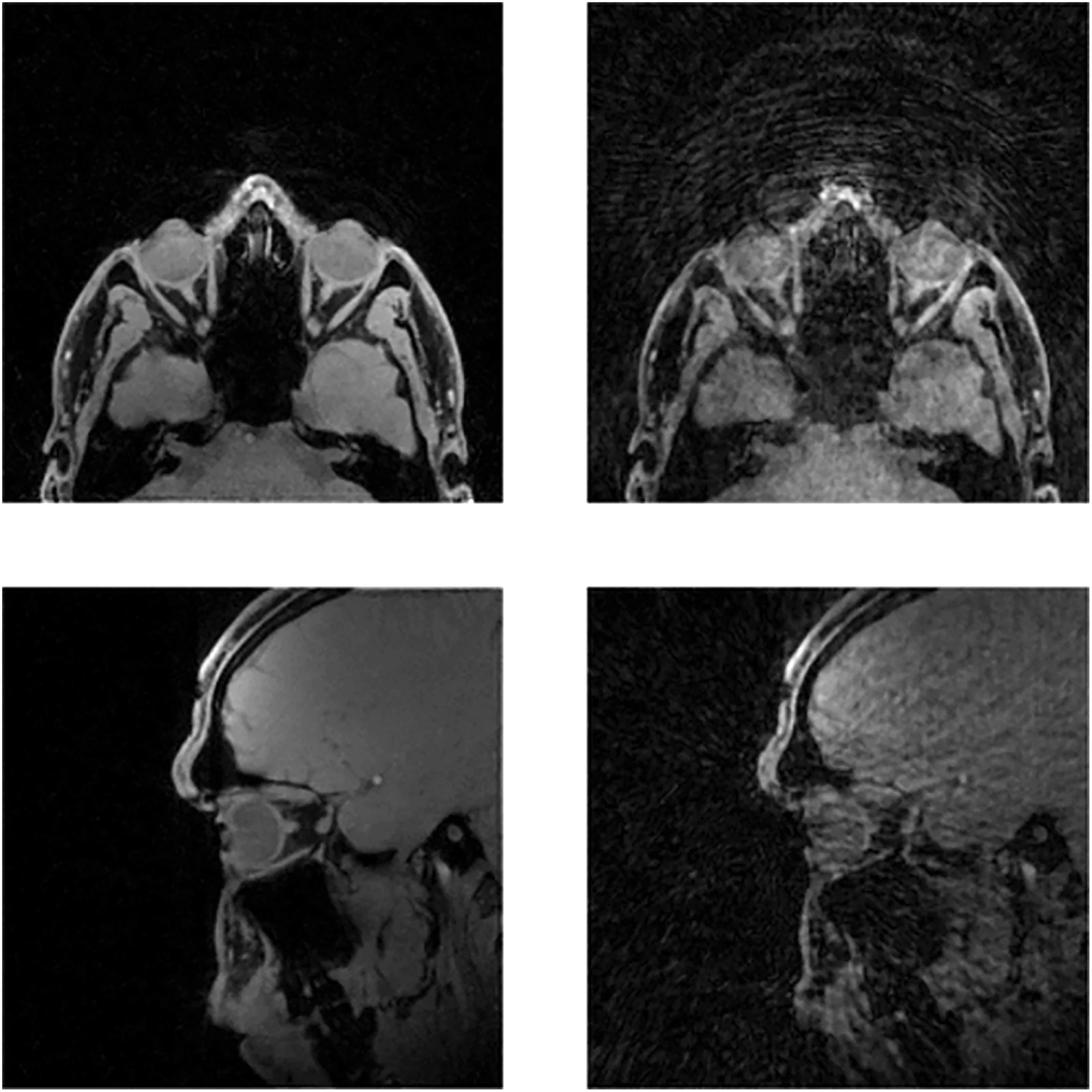
Comparison of images when binned based on eye position versus when binned consecutively (Left). Standard volume reconstructed using our technique. Top and bottom rows show an axial and sagittal view, respectively. (Right) When volumes are binned together based on continuously acquired k-space readouts, motion artefacts are apparent. Information corresponding to different eye positions across time is pooled together. This situation approximates the classical motion and under-sampling artefacts we would obtain while acquiring a T1-weighted image and reconstructing it without a motion-resolved compressed sensing algorithm. Reproduced from Franceschiello et al.^1^ with permission from Elsevier (Progress in Neurobiology).

As practical benchmarks for users implementing the protocol, a successful acquisition and reconstruction should reproduce the following performance indicators: strong monotonic agreement between eye-tracker–measured and MR-derived eye rotation (Spearman’s ρ ≈ 0.92–0.97 horizontally and ≈ 0.86–0.98 vertically, p < 0.001); agreement between MRI-derived and biometric axial length within ∼0.5 mm on average (mean absolute difference 0.48 ± 0.34 mm right eye, 0.55 ± 0.43 mm left eye; Spearman’s ρ = 0.92 and 0.77); more than ∼5,000 k-space readouts preserved after eye-motion binning (this will depend on the number of visual stimuli points presented on the screen); and a reconstruction time of ∼1 h per subject on adequate hardware (see previous section data processing). Users can additionally compute relative image-quality indices (SNR and CNR versus orbital fat) on the LIBRE volumes as a within-study check of reconstruction quality and fat suppression; in our data these were of order SNR ≈ 2.5–3.8 across ocular structures and CNR ≈ 2.5–2.7, though absolute magnitudes depend on background placement and normalization and are best interpreted relative to a user’s own baseline (see *Note*: Objective post-reconstruction quality metrics in data quality control section).

## Quantification and statistical analysis

### Quantitative assessment of motion tracking

The eye-tracker trajectories were used to reconstruct the volumes corresponding to different positions of the eyeballs, while the target position of the visual stimuli was used to compare the rotation extracted by the optometrist from the MR images. The projector screen was located 102 cm from the head coil, and the projected grey dots were positioned on a 4 × 4 grid with an average horizontal distance of 8.13 cm and a vertical one of 6.2 cm. This translates to an expected average rotation of the eyeball of about 4.55° in the horizontal direction and 3.47° in the vertical direction of motion. Among the 16 presentation positions, the top-left one was selected as the reference angle; and the difference with the other 15 positions was calculated. This difference allows for determining how well the proposed technique enables the correct estimation, from the motion-resolved reconstructed images, of the eyeball angular rotation during stimulation. For each participant and each direction of rotation, we calculated non-parametric correlation between the estimated rotation in the reconstructed MRI volumes and the rotation of the eye measured by the eye-tracker using Spearman’s rho (scatterplots in Figure 6). In the horizontal direction, these values ranged from 0.92 to 0.97 across participants and were each statistically reliable (p < 0.001 in each case). In the vertical direction, these values ranged from 0.86 to 0.98 across participants and were each statistically reliable (p < 0.001 in each case). These results indicate the capacity of our approach to track successfully both horizontal and vertical eye motion at an imaging level.

**Figure 6:**
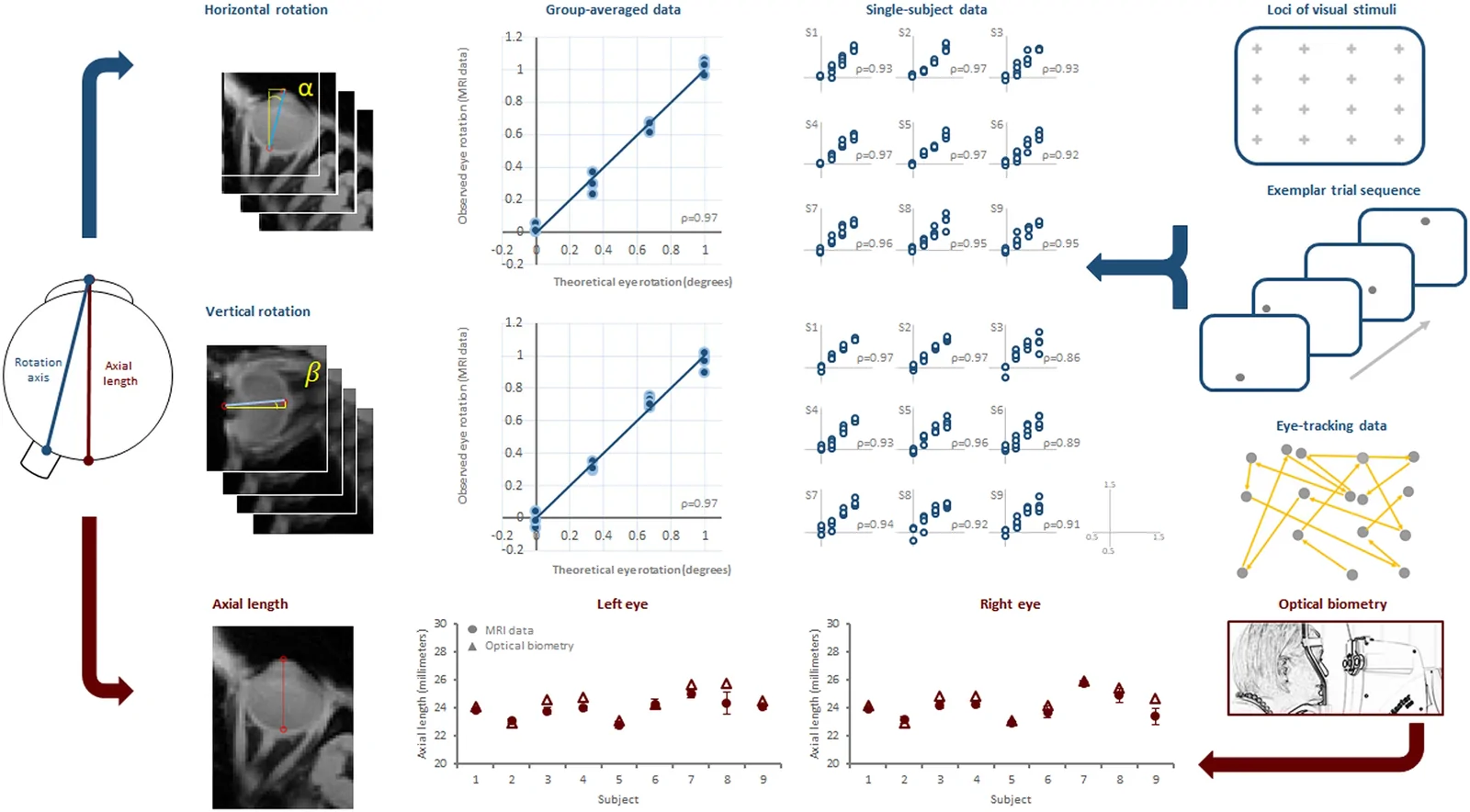
Quantitative validation of image quality Two classical benchmarking techniques were used to quantitatively assess not only the resolution of the images obtained from the protocol, but also their informativeness. First, the eye-tracker assessed motion changes (blue). Second, optical biometry evaluated the axial length of the eye (red). In the top part of the image we observe how the α, β – rotation angles estimated from the MR-images, linearly correlate (at a mean and individual subject level; Spearman’s rho indicated) with the theoretical rotation performed by the eye during the visual stimulation (both axes represent normalized differences in degrees). In the bottom part of the figure, it is shown how the axial length estimated across all presentations from the MR-images has the same order of magnitude (mm) of those estimated by the biometry (μ m accuracy). Reproduced from Franceschiello et al.^1^ with permission from Elsevier (Progress in Neurobiology).

### Quantitative assessment of eye image quality

All the MRI participants underwent an ocular biometry (IOLMaster 700, Zeiss) to enable us to estimate the axial length of each eye.^16^ In clinical practice, axial length is a principle diagnostic tool for myopia. We also estimated the axial length from the reconstructed MRI data (performed by a trained optometrist). The lower portion of Figure 6 displays these measurements for each participant and eye. The mean difference (± standard deviation) between these measures was 0.48 ± 0.34 mm for the right eye and 0.55 ± 0.43 mm for the left eye. Across individuals, the MRI-based and biometric estimates of the eye’s axial length were strongly correlated for each eye (Spearman’s rho = 0.92 and 0.77 for the right and left eye, respectively; p < 0.016 in both cases). This pattern undoubtedly proves that the technique, once optimized, could provide information about anatomical structures consistent with those obtained through classical optometric methods.

## Limitations

While the protocol offers a novel approach to motion-resolved acquisition without a fixation requirement, its adoption entails several practical dependencies related to hardware, synchronization, computational resources and participant population. These are outlined in the following paragraphs, together with the strategies that can mitigate them.

The protocol depends on hardware that is not part of a standard MRI installation: an MR-compatible eye tracker (here an EyeLink 1000 Plus with a 50 mm MR-compatible lens), a dedicated infrared mirror mounted on the head coil, and a hardware synchronization box. The infrared mirror is a delicate component, and setup and eye-tracker calibration add approximately 10–15 min per session, depending on operator expertise in camera positioning and focusing. Accurate, scanner-locked triggering is equally essential, as minor (few-millisecond) delays between the SyncBox, the scanner and the eye tracker can slightly mismatch the binning of MRI readouts to gaze position, and synchronization settings should be re-verified after scanner software upgrades; manual synchronization remains possible as a fallback, by extracting the physiological signal, identifying the timestamp of the first trigger, and aligning it to the moment the eye-tracking system starts recording. As with many imaging protocols, spatial and temporal resolution must also be balanced: increasing the number of readouts, expanding the field of view or achieving finer resolution extends acquisition time and thus increases susceptibility to motion artifacts, although careful protocol optimization can limit this effect.

Downstream, the motion-resolved compressed-sensing reconstruction is memory- and time-intensive (≥256 GB RAM, ∼1 h per subject) and typically requires a workstation or HPC cluster rather than the scanner console. Finally, although removing the fixation requirement helps participants who cannot fixate steadily, the protocol still requires that they tolerate the scanner environment, keep their head reasonably still, and follow (or at least freely explore) the visual targets. Young children and patients with severe nystagmus, ptosis, very poor vision or claustrophobia may remain difficult to image, and pupil detection can be degraded by droopy eyelids, mascara or eye dryness.

A further limitation is that the current implementation tracks only the right eye (monocular). Many ophthalmological and neuro-ophthalmological applications, however, require assessment of binocular coordination (e.g., strabismus, vergence, or internuclear ophthalmoplegia). Extending the protocol to binocular motion-resolved imaging is feasible in principle but would require setting up the eye tracker to track both eyes, binning of the k-space readouts against both gaze traces, and a reconstruction that jointly exploits the two eyes’ motion states; we regard this as an important direction for future development.

Finally, the protocol described here (DEBI) is the foundational implementation, and two derivative protocols extend it for different scenarios. MR-Eye Track focuses on eye-motion tracking and reconstruction using the fixation-shape target and a vendor-agnostic, open-source acquisition (T1w-LIBRE implemented in Pulseq) with reconstruction in the open-source Monalisa toolbox, lowering the barrier to adoption on non-Siemens systems. 2.0 MR-Eye extends the framework to multi-contrast ocular imaging (adding T1w-VIBE, T2w-LIBRE and T2w-TSE acquisitions) for richer anatomical characterization with central fixation. Both derivatives inherit the eye-tracking–driven, motion-resolved principle of DEBI but improve, respectively, portability/openness and contrast versatility; the choice among them depends on whether the priority is gaze classification (MR-Eye Track) or fixed central fixation with comprehensive multi-contrast assessment (2.0 MR-Eye). The corresponding datasets, code and step-by-step SOPs are referenced in the data and code availability section.

## Troubleshooting

### Problem 1

The visual stimuli experiment fails to run on the on-site laptop due to a conflict of version. This likely occurs because different developers use different *PsychoPy* versions during development. Related to steps 1, 15 and 16 in before you begin, and step 3 in step-by-step method details.

### Potential solution

• Ensure a consistent *PsychoPy* version across all platforms (development and execution) to prevent compatibility issues with the.psyexp file.
• Instead of running the psyexp file directly, execute the generated Python script (.py) from *PsychoPy* in the terminal within the *PsychoPy* environment for greater stability.
• Use Git to track both the experiment files and *PsychoPy* configurations to maintain version control.

### Problem 2

The calibration or validation procedure of the eye-tracker is rejected. Related to steps 17, 18 and 19 in before you begin.

### Potential solution

• Ensure the participant’s forehead is firmly pressed against the head coil before entering the MRI scanner. If needed, use sponge pads to adjust head height.
• Turn off the scanner’s interior light to prevent interference with the eye-tracker’s infrared reflection system.
• Verify that the eye-tracker camera properly focuses on the pupil, with eyelashes visible in detail. If not, adjust the lens to the recommended setting (around 1) and fine-tune the smaller knob for precise focus.
• Avoid any type of possible source of artefacts, such as make-up or facial creams.

### Problem 3

The participant can become fatigued during acquisition, keeping their eyes closed for extended periods. This results in a high proportion of non-applicable data in eye-tracking recordings, degrading data quality, and impacting motion-resolved reconstruction. Related to the “Image acquisition” steps and to “Data quality control”. Related to steps 1, 2, 3 and 14 in step-by-step method details.

### Potential solution

• Monitor the participant’s eye status in real time on the eye-tracking (ET) PC.
• If the participant is falling asleep, use the microphone to check their wellbeing.
  ○ If the participant feels unwell or too fatigued, stop the scan and guide them out of the scanner, rescheduling if necessary.
  ○ If the participant is simply tired but willing to continue, provide gentle encouragement to follow the stimuli while ensuring their comfort.

### Problem 4

The participant cannot see the full visual stimuli in the MR-Eye Track protocol, especially dots near the top, bottom, left, or right edges. Related to steps 8, 9, 11, 12, 17, 18, 19 in before you begin.

### Potential solution

Ensure the monitor resolution is set to 800×600. If left at the default (e.g., 1920×1080), the projection onto the half-circle screen behind the scanner may be misaligned, causing stimuli near the edges to fall outside of the visible area to the participant.

### Problem 5

Visual stimulation has started, but not the scanning or vice versa. Related to the SyncBox setup steps in “preparation outside the scanner room” and to “activate the EXT trigger” in Image acquisition. Related to steps 10 and 11 in before you begin, and step 3 in step-by-step method details.

### Potential solution

Ensure that the EXT trigger cable is connected to the scanner and to the Sync Box, and that it’s also connected inside and outside the scanner room to the same channel port. Check that in the IDEA UI, the EXT trigger is enabled in the Physio section.

### Problem 6

There are no EDF files generated or they are empty. (Related to the “Session completed” and “Collect ET data” steps in Session tear-down.) Related to step 8 in step-by-step method details.

### Potential solution

Ensure that there is a line in your *PsychoPy* code to start the ET recording (usually *etRecord.start()*) and check that in the ET’s PC there is a textbox with an increasing number, this indicates the samples recorded by the ET, and it means it is recording correctly. It is mandatory to wait until the end of the experiment to allow the transfer of the EDF file and do not interrupt this process by disconnecting the cable, it may take several seconds. See https://mattechlab.github.io/sops/debi/data-collection/2_run_scanning_session/#session-completed.

## Resource availability

### Lead contact

Further information and requests for resources should be directed to the lead contact, Benedetta Franceschiello (benedetta.franceschiello@hevs.ch).

### Technical contact

Technical questions on executing this protocol should be directed to and will be answered by the technical contacts, Jaime Barranco (jaime.barrancohernandez@hevs.ch), Yiwei Jia (yiwei.jia@hevs.ch), and Benedetta Franceschiello (benedetta.franceschiello@hevs.ch).

### Materials availability

This study did not generate new unique materials or reagents. The datasets supporting the original DEBI study have not been deposited in a public repository but are available from the corresponding author on reasonable request.

### Data and code availability

• The accession number for the MR-Eye Track MRI and eye-tracking data is OpenNeuro: ds008973 [DOI: https://doi.org/10.18112/openneuro.ds008973.v1.0.0]. The code is available at https://github.com/MattechLab/MR-EyeTrack.
• The *PsychoPy* experiment program used to display the visual stimuli is available at (from the derivative study MR-Eye Track) https://github.com/MattechLab/MR-EyeTrack/blob/dev/visual_stimuli/mreyetrack_16points.py.
• The Python code for post-processing of eye-tracking data and generation of the binning mask for motion-resolved reconstruction is available at https://mattechlab.github.io/sops/debi/data-processing/1_2_et_QAQC_of_mreye_tracking/.
• The MATLAB code for motion-resolved reconstruction is implemented in the open-source Monalisa toolkit,^13^ available at https://doi.org/10.1101/2025.06.06.658299, with full documentation at https://mattechlab.github.io/monalisa/index.html.
• Additional standard operating procedures (SOPs), including step-by-step data processing guides for the DEBI protocol and its derivatives, are available on the MatTechLab SOP webpage: https://mattechlab.github.io/sops/.

## Acknowledgments

Financial support for this work was provided by the Fondation Asile des aveugles (grant #232933 to M.M.M.), a grantor advised by Carigest SA (#232920 to M.M.M.), as well as the Swiss National Science Foundation (grants #169206 to M.M.M. and #220433 and #229214 to B.F.) and the HES-SO University of Applied Sciences and Arts Western (grant 129816/IA-RECHERCHE23-19). We thank all the coauthors for their earlier contributions to this work.

## Author contributions

For the current protocol paper, J. Barranco, B.F., Y.J., and M.M.M. conceptualized and documented the protocol. M.L., J. Bastiaansen, H.V., E.F., J.-B.L., and O.E. contributed to the implementation of the derivative study 2.0 MR-Eye (described in the data and code availability section). C.C.C., E.N., and L.E.R. contributed to the implementation of the derivative study MR-Eye Track.

## Declaration of interests

The authors had a previous patent application related to the original study, but this patent has now expired. Therefore, the authors declare no competing interests.
